# Knowledge map of recurrence in tenosynovial giant cell tumor: a bibliometric analysis

**DOI:** 10.3389/fonc.2025.1523979

**Published:** 2025-11-05

**Authors:** Pan Gao, Shuo-Cheng Ji, Wei Liang, Guang-Jian Bai, Jia-Ji Ren, Fu-long Zhong, Long Yang, Jing Wang, Bao-Quan Xin, Tie-Long Liu

**Affiliations:** ^1^ Anhui University of Science & Technology, Medical School, Huainan, China; ^2^ Department of Orthopaedic Oncology, Changzheng Hospital of the Navy Medical University, Shanghai, China; ^3^ Department of Orthopedics, Shanghai 411 Hospital, Affiliated Hospital of Shanghai University, Shanghai, China; ^4^ University of Shanghai for Science and Technology, School of Health Science and Engineering, Shanghai, China; ^5^ School of Clinical Medicine, Shandong Second Medical University, Weifang, Shandong, China

**Keywords:** bibliometric analysis, bone tumor, radiotherapy, tenosynovial giant cell tumor, recurrence, surgical treatment

## Abstract

**Purpose:**

Tenosynovial Giant Cell Tumor (TGCT) is a rare benign neoplasm originating from the synovium and tendon sheath, and is characterized by a relatively high rate of recurrence following treatment. The present study was designed to identify and analyze 134 publications in this domain, with the aim of providing researchers with a comprehensive overview of the knowledge framework and research hotspots related to TGCT recurrence.

**Methods:**

A total of 134 articles concerning TGCT recurrence were retrieved from the Web of Science Core Collection. Bibliometric analysis was applied to examine various characteristics of the literature. VOSviewer and Microsoft Excel were employed to perform visualizations of temporal and geographical distributions, author productivity, thematic classifications, topic evolution, reference networks, and keyword co-occurrence.

**Results:**

Among the identified articles, the most frequently cited publication received 123 citations. The University of California was found to be the most influential institution in terms of citation frequency, while the United States ranked first in the number of publications. A steady increase in research activity was observed, particularly between 2021 and 2024. Nicholas Matthew Bernthal was identified as the most prolific author, with eight publications. Keywords indicating major research hotspots included “tenosynovial giant cell tumor,” “surgical treatment,” “recurrence,” “synovectomy,” and “radiotherapy.” Fourteen articles specifically addressed multimodal treatment strategies, highlighting a critical direction of research in this field.

**Conclusion:**

Research on TGCT recurrence has received increasing attention, with particular emphasis on multimodal treatment strategies. Nevertheless, the underlying biological mechanisms remain insufficiently elucidated. Further exploration of these mechanisms is warranted to enable future therapeutic advances.

## Introduction

Tenosynovial giant cell tumor (TGCT), also known as pigmented villonodular synovitis (PVNS), represents a group of benign lesions sharing common pathogenesis and histopathological features. The disease originates from the synovium, tendon sheath, and bursae, and is characterized by synovial proliferation and hemosiderin deposition (1). According to the World Health Organization (WHO) Classification of Soft Tissue and Bone Tumors (2013), TGCT can be classified into localized and diffuse types. The incidence rates of localized and diffuse TGCT are 30.3 and 8.4 per million population per year, respectively. For localized TGCT, the ten-year postoperative recurrence risk is as high as 8.9%, with a lifetime recurrence rate of 15%; in contrast, diffuse TGCT exhibits a ten-year recurrence rate of 15.5% and a lifetime recurrence rate of up to 55% (2). Recurrent disease significantly impairs patients’ quality of life and imposes a substantial burden on both families and society (3–5).

As research on TGCT recurrence has progressed, the number of related publications has steadily increased, creating challenges for researchers in efficiently identifying and synthesizing relevant literature. Bibliometrics, a key branch of information science, focuses on the quantitative and qualitative analysis of literature, emphasizing the characteristics and metrics of the publication system. This approach enables the quantification of distribution patterns, associations, and clustering within a research domain, and has become an essential tool for evaluating the credibility, quality, and impact of academic output (6–10). To date, no dedicated bibliometric studies have been conducted on TGCT recurrence, and systematic, high-precision analyses of this topic remain lacking.

To address this knowledge gap, a comprehensive bibliometric analysis of all published literature on TGCT recurrence was performed. The study encompassed publication year, journal type, author information, institutional affiliations, country of origin, keyword occurrence, and citation patterns. Data visualization techniques were applied to provide an intuitive overview of current research trends and developmental directions in the field. This study aims to advance TGCT recurrence research, provide scholars with timely insights, reduce the time and effort required for literature retrieval, and offer researchers, clinicians, and policymakers a comprehensive summary of the latest developments in this domain.

## Methods

The Web of Science (WoS) is a globally recognized research platform, covering vast information resources across science, arts, and humanities, and is widely trusted by international publishers as an independent and authoritative citation database. To ensure the representativeness and accessibility of data, all publications related to recurrence of TGCT were retrieved from the WoS database. It should be noted that TGCT has long been referred to by most orthopedic surgeons as PVNS. Although the World Health Organization revised the nomenclature over a decade ago, a substantial portion of the literature continues to use the term PVNS. To minimize retrieval bias, both “TGCT” and “PVNS” were included as search keywords. Specifically, the search was conducted in the title field using “tenosynovial giant cell tumor” OR “PVNS,” and in the topic field using “recur*” OR “relapse,” while literature types such as letters, conference papers, and patents were excluded.

VOSviewer (version 1.6.20) is a bibliometric software widely used to extract key information from large volumes of publications and to construct networks of collaboration, co-citation, and co-occurrence (11). In the present study, VOSviewer was primarily employed for analyses of countries and institutions, journals and co-cited journals, authors and co-cited authors, and keyword co-occurrence. In the visualized maps generated by VOSviewer, nodes represent entities such as countries, institutions, journals, or authors. The size and color of nodes indicate the quantity and classification of these entities, while the thickness of links reflects the degree of collaboration or co-citation relationship.

**Figure 1:**
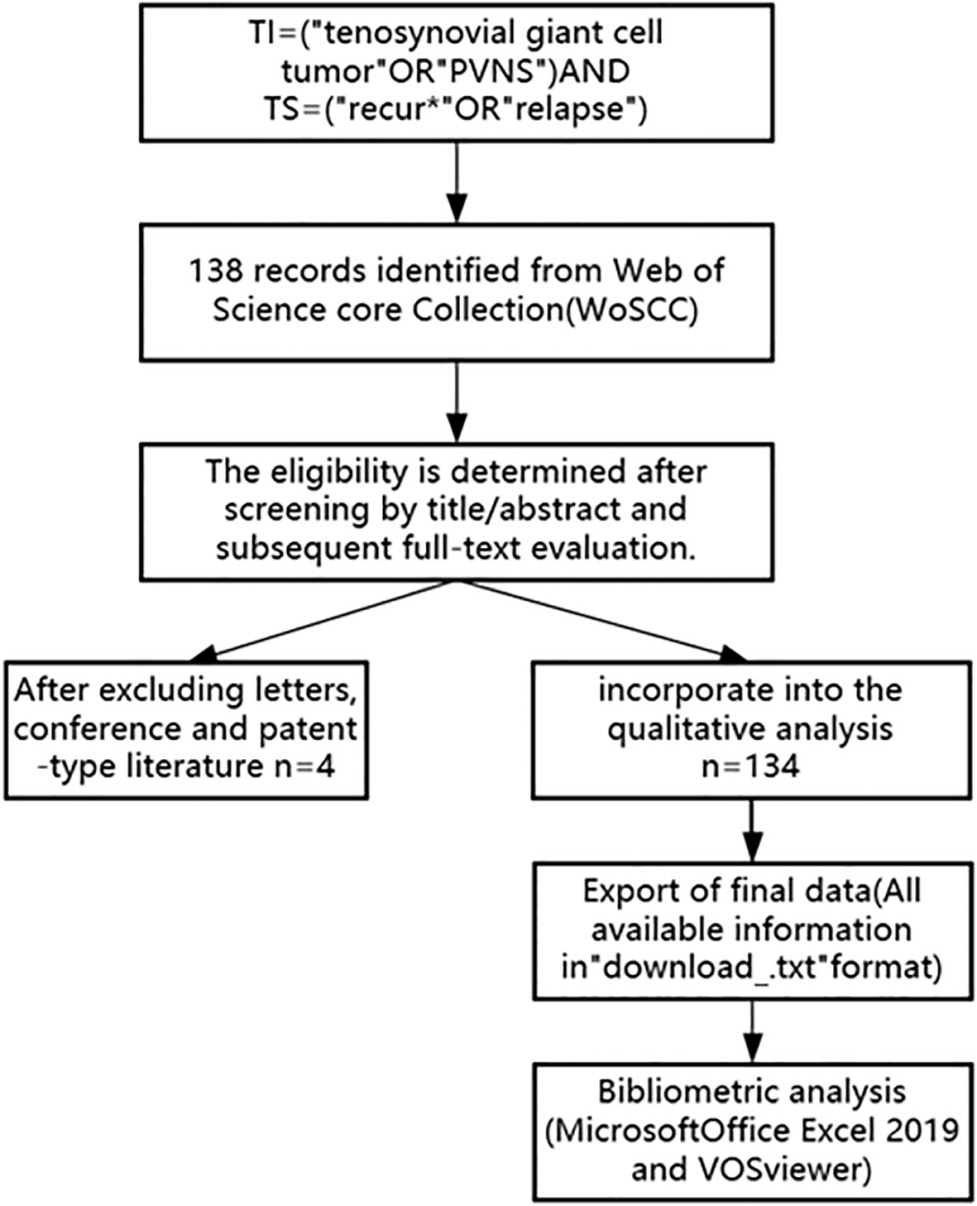
Publications screening flowchart (the flowchart illustrates the strategy and selection process for TGCT recurrence literature).

Based on the search strategy, a total of 138 records were retrieved as of September 2025. Three independent reviewers screened the titles and abstracts, followed by full-text assessment, excluding two letters, one conference paper, and one patent, resulting in a final dataset of 134 relevant publications (**Figure 1**). The reviewers independently categorized the literature, and discrepancies were resolved through discussion to reach consensus. Key elements were recorded and analyzed, including article title, authors, publication year, journal name, total citations, average citations per year, country, institution, and keywords. VOSviewer was used to visualize the distribution of these key elements.

Upon detailed examination of the methodologies employed in the collected literature, studies were classified into three categories based on research design: reviews, clinical studies (diagnosis, treatment, and prognosis based on clinical data), and basic research (addressing various disease-related issues from a fundamental scientific perspective). These publications were further subdivided into seven major themes: surgical treatment-related research, non-surgical treatment-related research, multimodal treatment strategies, diagnosis and feature analysis of TGCT in the head and neck, prognostic factor analysis, exploration of TGCT pathogenesis, and case reports.

## Result

**Figure 2:**
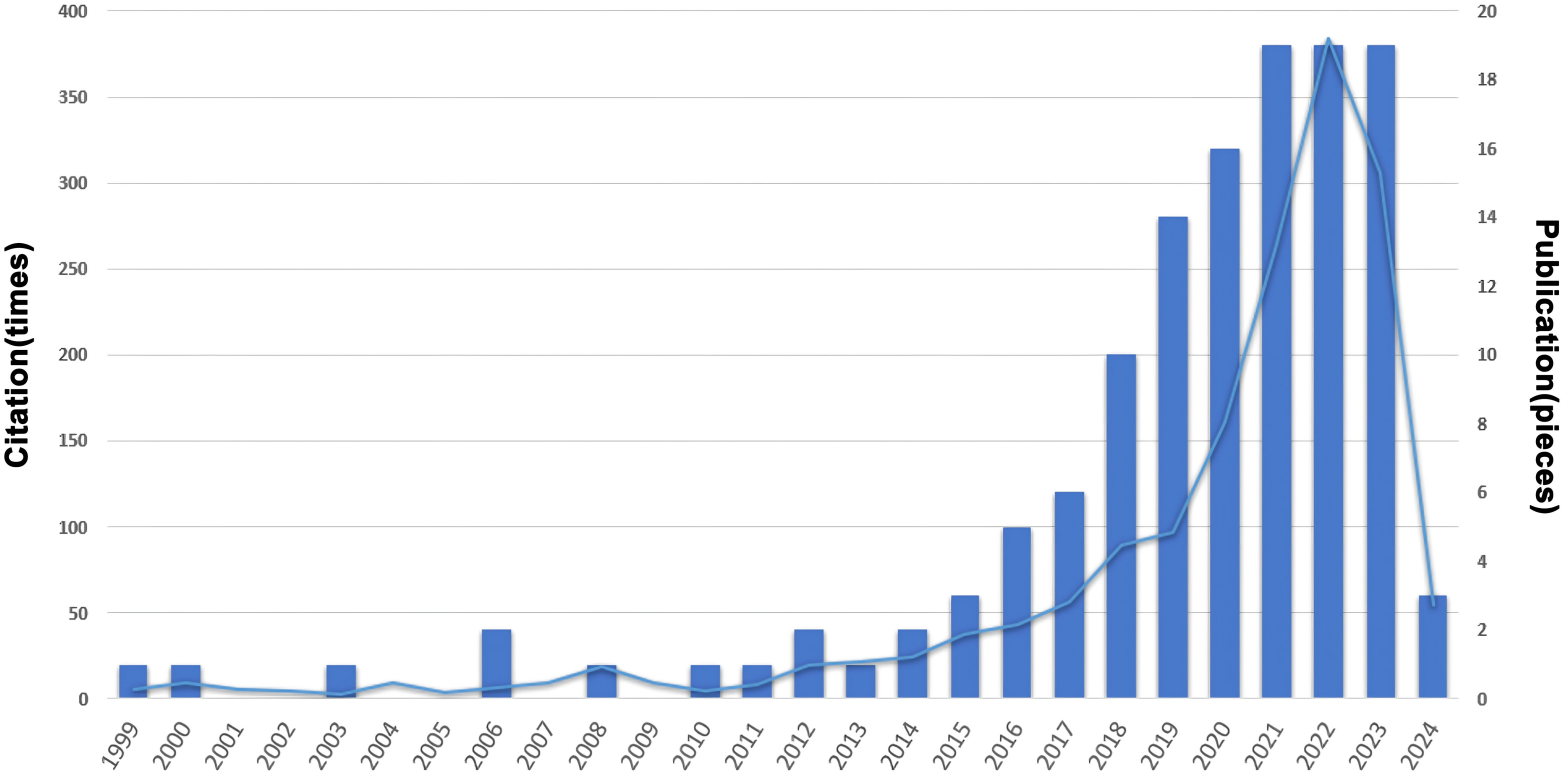
Trends in the growth of publications and the number of ciations.

A total of 134 publications on TGCT recurrence were retrieved according to the established search criteria. These articles accumulated 1,431 citations, with the most-cited paper receiving 123 citations and an average of 10.7 citations per article. The highest average annual citation rate was 20.5, corresponding to a review published in 2020. The largest number of publications occurred in 2021, with 22 articles, indicating a steady growth in research output in this field (**Figure 2**).

**Figure 3:**
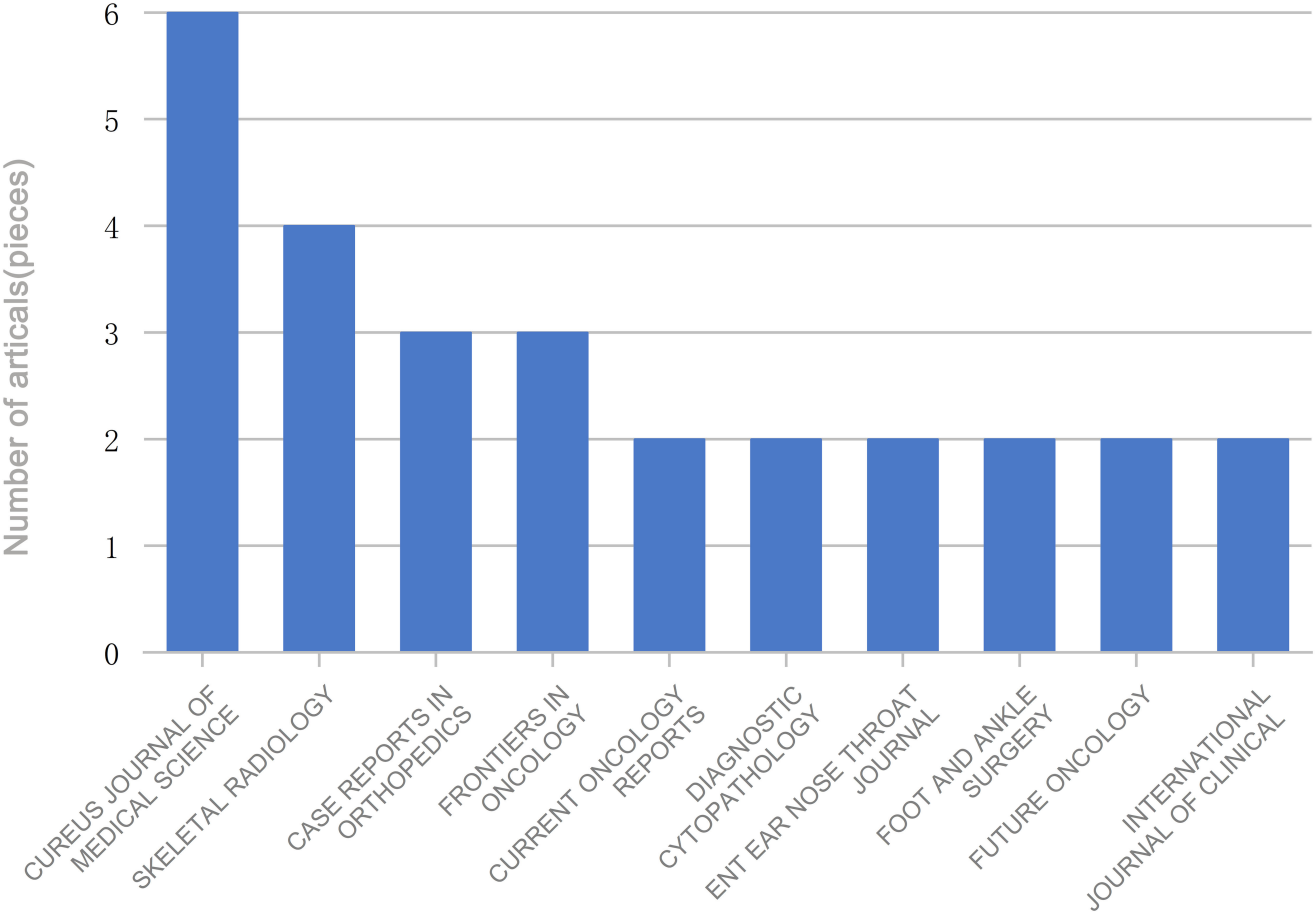
Journals with multiple publications on TGCT recurrence.

**Figure 4:**
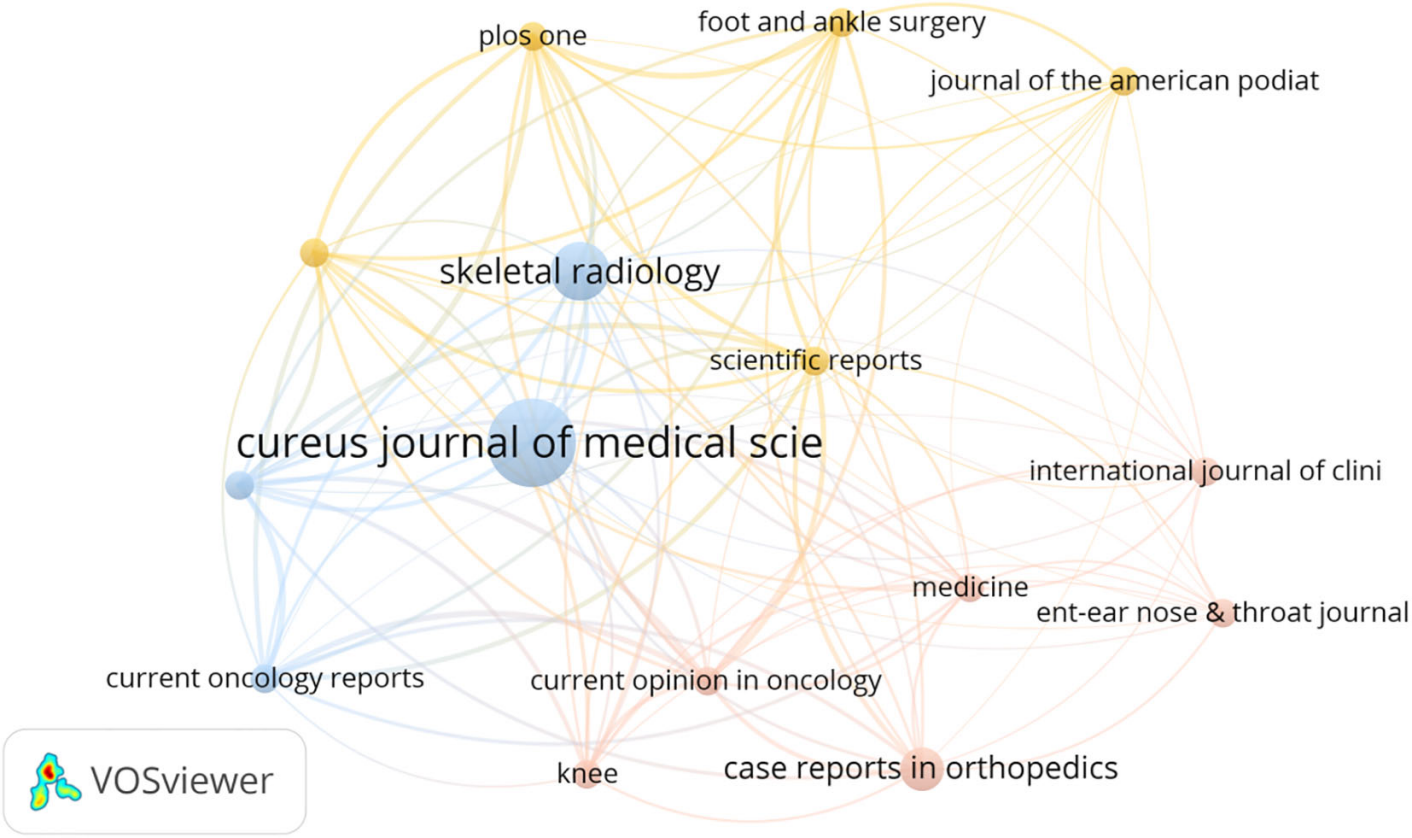
The journals collaboration network of the most cited articles on TGCT recurrence. From: VOSviewer.

**Figure 5:**
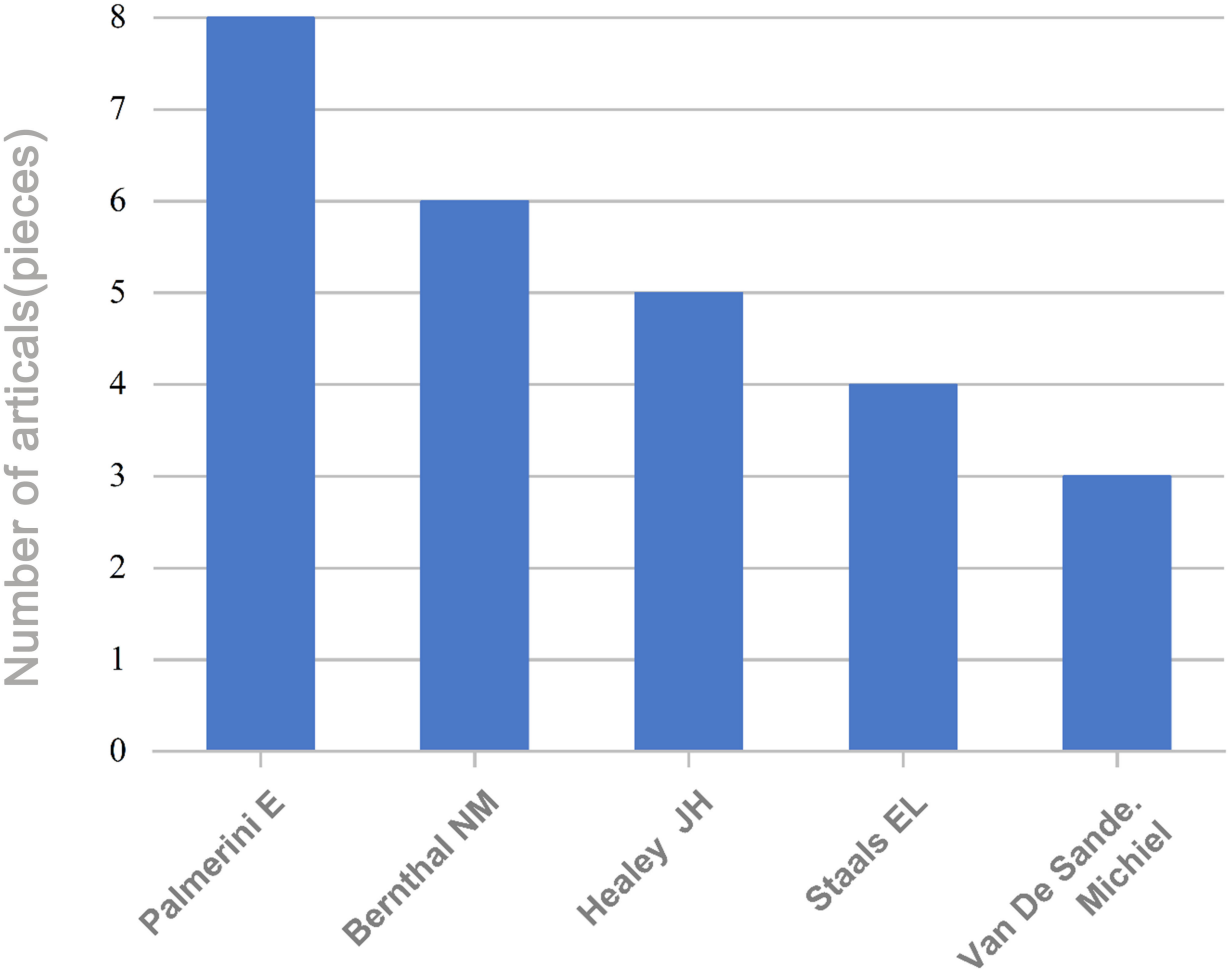
Authors with multiple publications.

**Figure 6:**
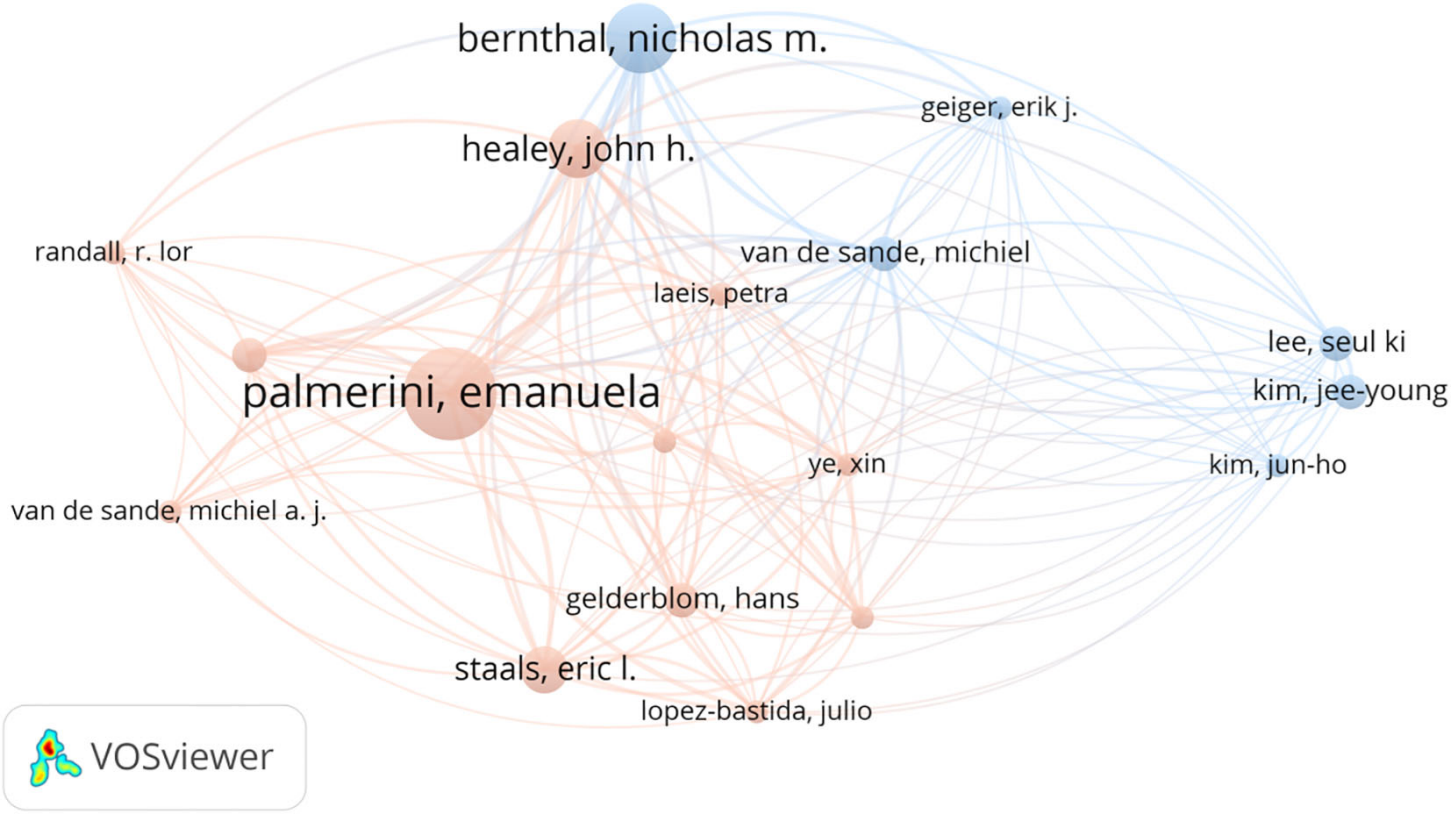
The authors collaboration network of the most cited articles on TGCT recurrence. From: VOSviewer.

The 134 articles were published across 100 journals. Cureus Journal of Medical Science contributed the largest number, with six publications, followed by Skeletal Radiology, Case Reports in Orthopedics, and Frontiers in Oncology, which contributed four, three, and three articles, respectively (**Figure 3**). Co-authorship networks of journals are shown in **Figure 4**. A total of 710 authors contributed to these studies. Palmerini emerged as the most prolific author with eight publications, followed by Bernthal NM, Healey JH, Staals EL, and Van De Sande, contributing six, five, four, and three articles, respectively (**Figure 5**). The co-citation network among authors is illustrated in **Figure 6**. Further analysis of the collaboration network revealed that Palmerini has made substantial contributions to understanding TGCT incidence, treatment outcomes, and prognostic factors, with eight first-author publications. The most-cited paper by Palmerini, Tenosynovial Giant Cell Tumor/PVNS: Prognosis of 294 Patients Prior to the Era of Kinase Inhibitors (12), has been widely recognized. Current mainstream TGCT treatment involves surgical excision combined with targeted therapy and chemotherapy, offering improved control of tumor recurrence compared to conventional approaches. Collaborative studies between Staals and Palmerini have explored novel therapeutic strategies and conducted multicenter prospective prognostic analyses, significantly advancing the field. While Healey, Van De Sande, and Staals were not first authors in all publications, their collective 18 articles underscore substantial academic contributions.

**Figure 7:**
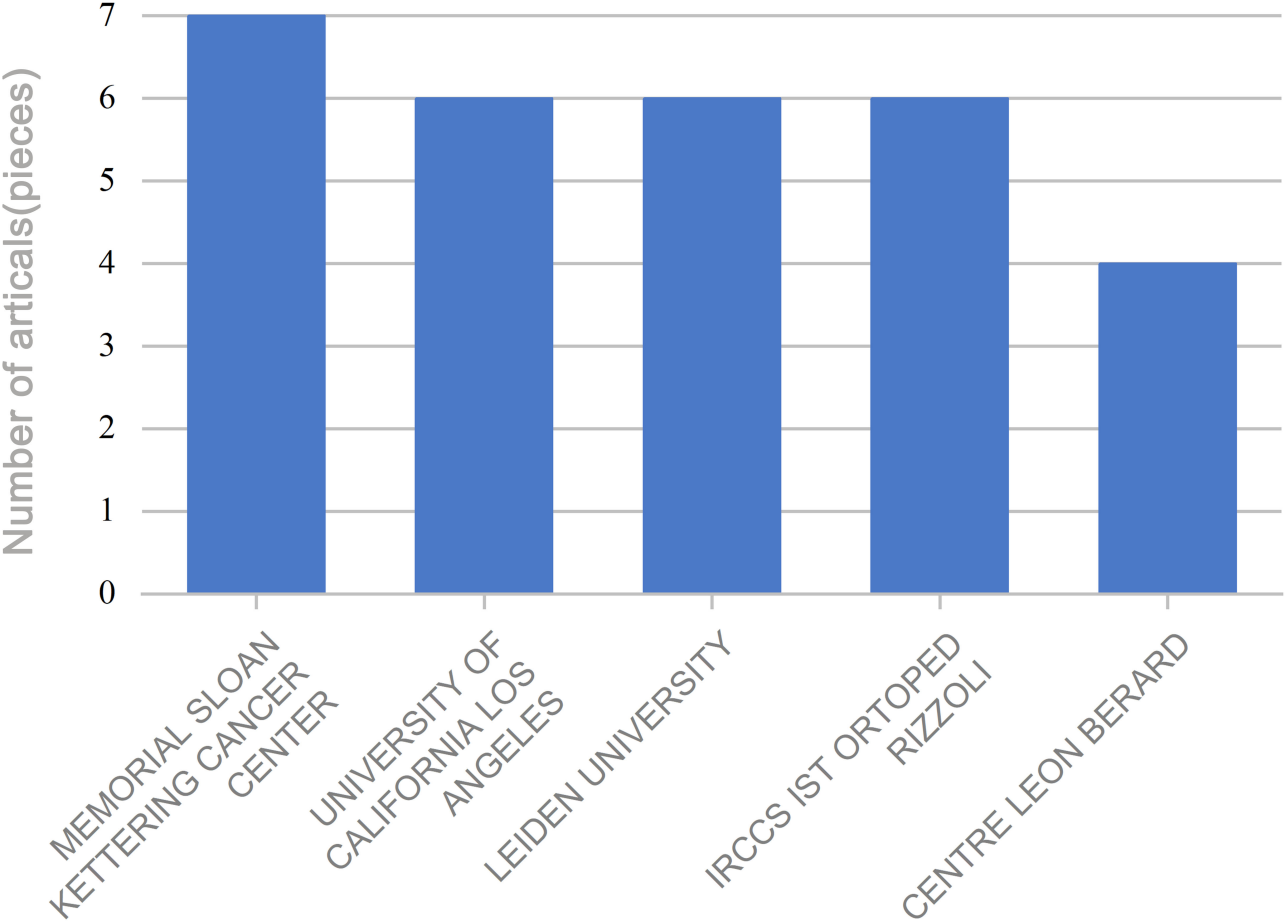
Institutions with multiple publications.

**Figure 8:**
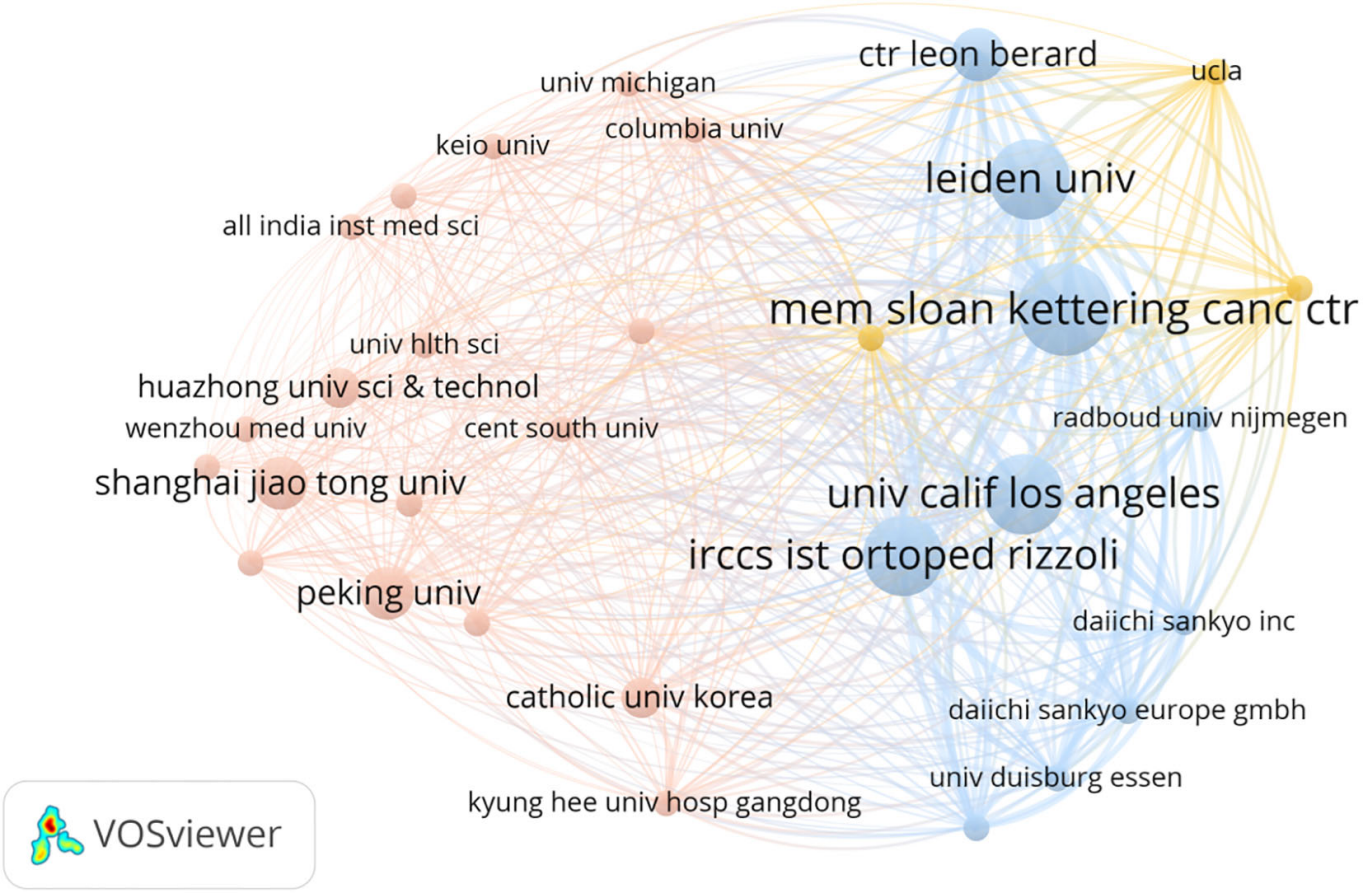
The institutions collaboration network of the most cited articles on TGCT recurrence. From: VOSviewer.

**Figure 9:**
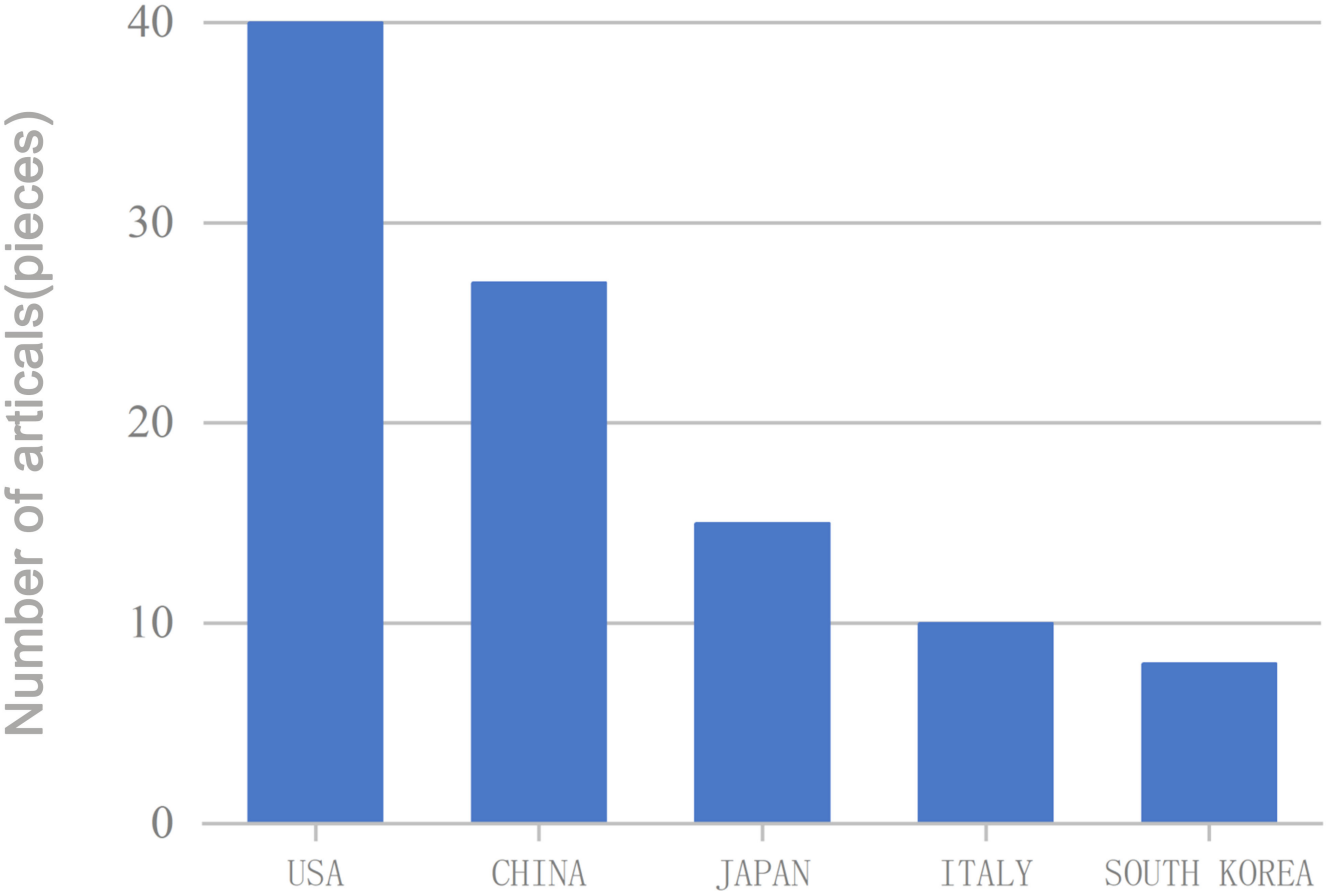
Countries with multiple publications.

**Figure 10:**
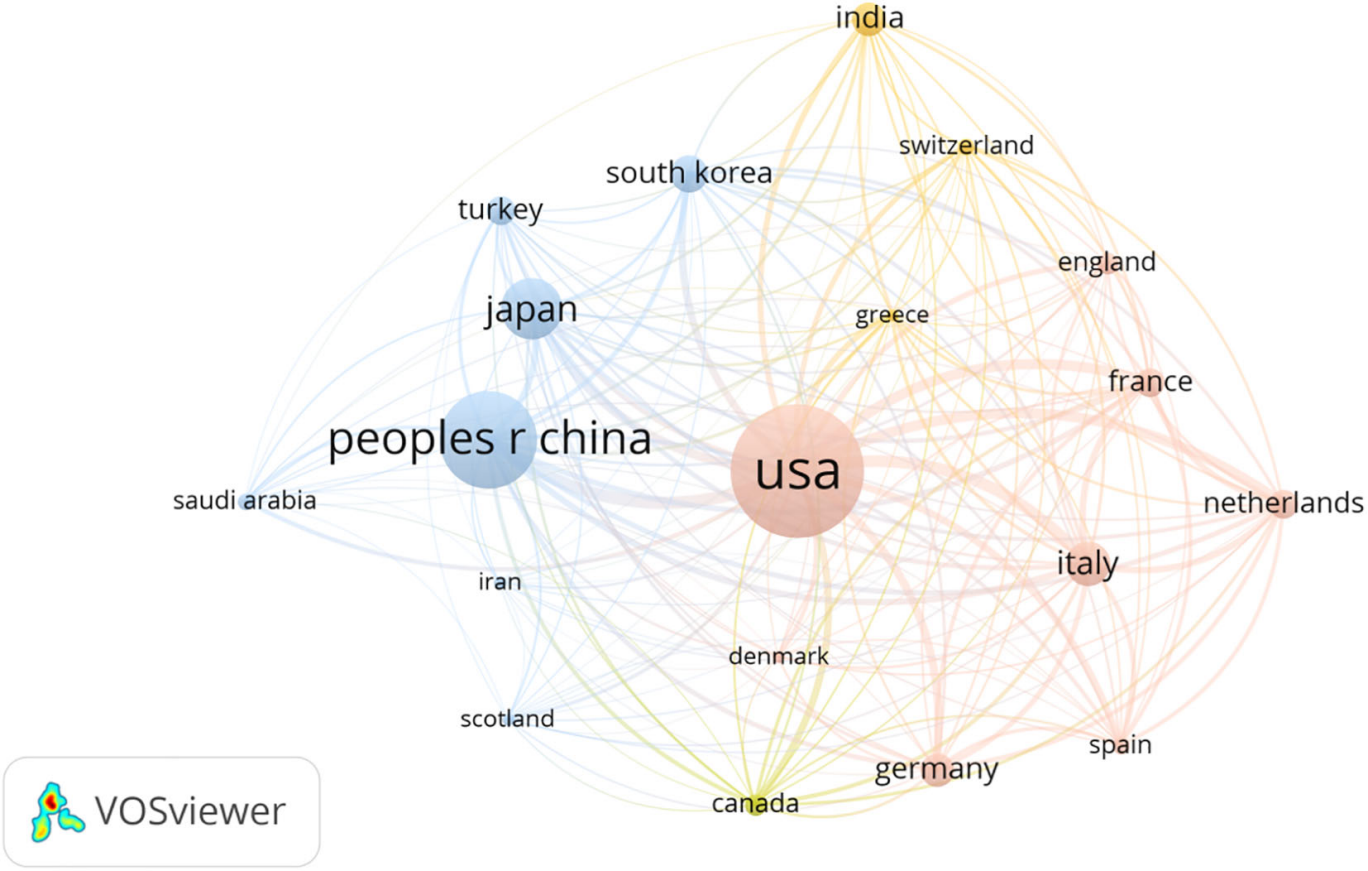
The countries collaboration network of the most cited articles on TGCT recurrence. From: VOSviewer.

The authors were affiliated with 100 institutions, of which 48 appeared multiple times. Memorial Sloan Kettering Cancer Center was the most frequently listed institution, appearing seven times (**Figure 7**). Institutional collaboration networks are shown in **Figure 8**. These institutions spanned 29 countries and regions, with 19 countries appearing multiple times. The United States led in publication count, number of researchers, and participating institutions, followed by China. The US accounted for 40 mentions, China 27, and Japan 15. Publications from the US and China together represented nearly half (50.0%) of all studies (**Figure 9**). International collaboration networks are shown in **Figure 10**.

**Figure 11:**
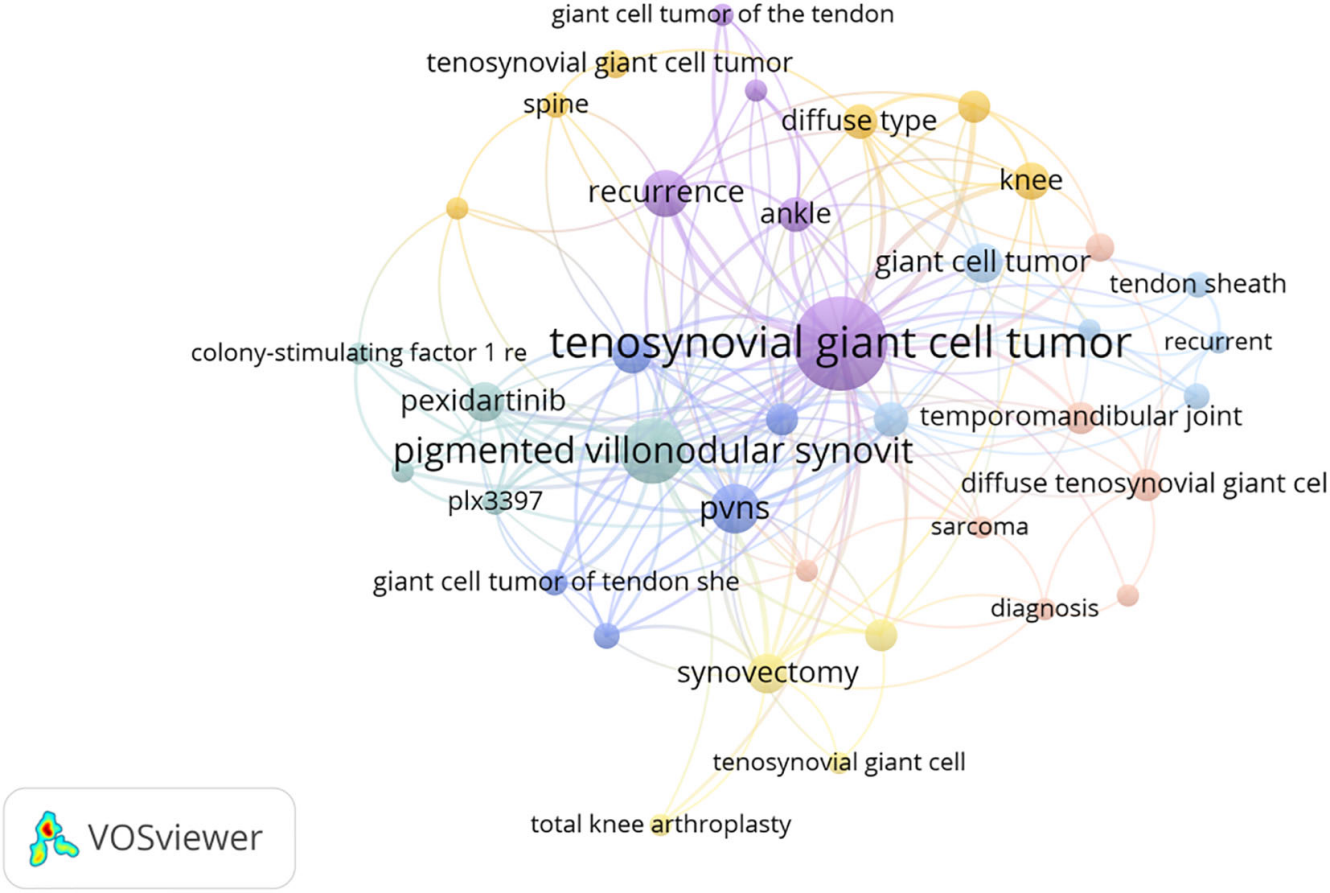
Cooccurrence overlay visual analysis of keywords that appear more than 2 times within the overall retrieved documents. The connections are keywords with a link strength >5. From: VOSviewer.

**Figure 12:**
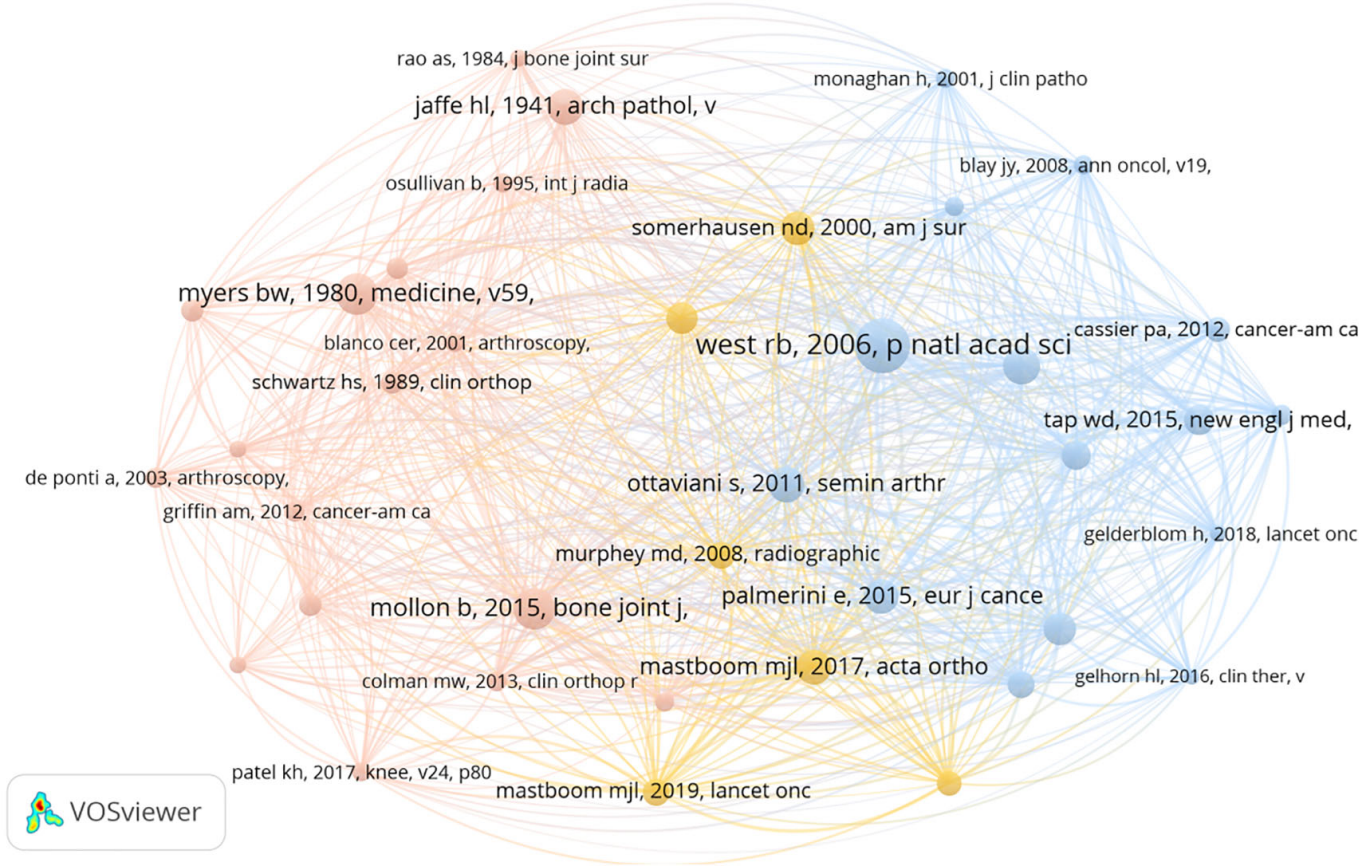
The reference cocitation network of the most cited articles on TGCT Recurrence. The connections are citations with a link strength >10. The largest nodes were from West rb (2006). From: VOSviewer.

Among 429 keywords, 15 reached a threshold of 10 occurrences, including “tenosynovial giant cell tumor,” “surgical treatment,” “recurrence,” “radiotherapy,” and “pexidartinib.” To ensure accuracy, singular/plural forms, synonyms, and abbreviations were standardized. Node size represents keyword frequency, while node distance reflects the strength of associations. The co-occurrence overlay map of all keywords is presented in **Figure 11**. Using a threshold of five occurrences, 44 keywords were analyzed. Co-citation analysis of references with a threshold of 13 included 38 studies(**Figure 12**), with the largest node derived from West RB (2006).

Based on study design, 100 studies were clinical, 12 were basic research, and 22 were reviews. Thematically, 45 publications focused on treatment, including 19 on surgical treatment, 12 on non-surgical interventions, and 14 on combined strategies. Additional analyses included 18 studies on prognostic factors, seven on TGCT pathogenesis and recurrence mechanisms, 17 on diagnostic techniques and tumor features, and 47 case reports. The most-cited paper among the 134 articles was Pexidartinib, a Novel Small-Molecule CSF-1R Inhibitor for TGCT: Systematic Review of Preclinical and Clinical Development (**Table 1**) (2, 13–21), published by Brooke Benner at the Ohio State University Comprehensive Cancer Center in 2020. This review highlighted that surgical excision remains the standard treatment for TGCT; however, recurrence, unresectable tumors, or potential limb amputation remain challenges. The review also emphasized that CSF-1/CSF-1R-targeted agents, such as pexidartinib, demonstrated significant efficacy, though long-term effects require further clinical follow-up (22, 23). The second most-cited article, Behavior and Global Expression Profiles Reveal Mechanistic Differences in Neuropathic Pain Types (19), published in Cell Reports by Boston Children’s Hospital in 2018, highlighted mechanism-based interventions for neuropathic pain following peripheral nerve injury, including targeting TrpV1-positive nociceptors and immune cell modulation. The third highly cited study, Localized and Diffuse Tenosynovial Giant Cell Tumor (Previously TGCT/PVNS) (16), published by F. Gouin from CHU de Nantes in 2016, reaffirmed complete surgical resection as first-line treatment, emphasizing more aggressive approaches for diffuse TGCT and the therapeutic potential of CSF-1R inhibitors.

**Table 1:** The 10 most cited articles among the 126 related papers.

Rank	Title	Journal	First author	Times cited, WoS core	Year
1	Pexidartinib, a Novel Small Molecule CSF-1R Inhibitor in Use for Tenosynovial Giant Cell Tumor: A Systematic Review of Pre-Clinical and Clinical Development	Drug Design, Development and Therapy	Brooke Benner (13)	123	2020
3	Mechanistic Differences in Neuropathic Pain Modalities Revealed by Correlating Behavior with Global Expression Profiling	Cell Reports	Enrique J. Cobos (16)	117	2018
2	Localized and diffuse forms of tenosynovial giant cell tumor (formerly giant cell tumor of the tendon sheath and pigmented villonodular synovitis)	Orthopaedics & Traumatology: Surgery & Research	F. Gouin (19)	116	2017
4	Does Combined Open and Arthroscopic Synovectomy for Diffuse PVNS of the Knee Improve Recurrence Rates?	Clinical Orthopaedics and Related Research	Matthew W. Colman (17)	66	2013
5	Tenosynovial Giant Cell Tumor: Incidence, Prevalence, Patient Characteristics, and Recurrence. A Registry-based Cohort Study in Denmark	The Journal of Rheumatology	Vera Ehrenstein (2)	64	2017
6	Long-term efficacy of imatinib mesylate in patients with advanced Tenosynovial Giant Cell Tumor	Scientific Reports	F. G. M. Verspoor (21)	46	2019
7	Management of Tenosynovial Giant Cell Tumor: A Neoplastic and Inflammatory Disease	JAAOS Global Research & Reviews	John H. Healey (20)	44	2020
8	The diffuse-type tenosynovial giant cell tumor (dt-TGCT) patient journey: a prospective multicenter study	Orphanet Journal of Rare Diseases	Nicholas M. Bernthal (15)	36	2021
9	Tenosynovial giant cell tumor: case report of a patient effectively treated with pexidartinib (PLX3397) and review of the literature	Clinical Sarcoma Research	Mastboom, Monique J. L.	34	2018
10	Management of Pigmented Villonodular Synovitis (PVNS): an Orthopedic Surgeon’s Perspective	Current Oncology Reports	Nicholas M. Bernthal (14)	33	2020

The most recent publication, Microsurgical Resection of Tenosynovial Giant Cell Tumor in the Digits (24), by K. Muramatsu, reviewed 34 patients treated with microsurgical excision, with a mean follow-up of 27.6 months and a recurrence rate of only 2.9%, the lowest reported to date. The study demonstrated that microsurgery allows precise visualization of tumor involvement in joint capsules, tendon sheaths, and bone, facilitating complete excision while preserving neurovascular structures, indicating its potential as the preferred treatment for digital TGCT. Conversely, the earliest study in the dataset, Scintigraphic Evaluation of Tenosynovial Giant-Cell Tumor Using Technetium-99m(V)-Dimercaptosuccinic Acid (25), published by Hisataka Kobayashi in 1993, reported three cases of primary and recurrent TGCT and demonstrated the utility of ^99mTc(V)-DMSA in lesion detection, whereas ^67Ga-citrate showed minimal uptake. These findings highlight a research evolution from imaging-based recurrence detection in early studies to surgical technique optimization in recent research.

## Disscussion

Our study Our analysis indicates that research on TGCT recurrence has predominantly focused on treatment strategies. Despite the therapeutic approach employed, local recurrence remains high for certain TGCT subtypes. Over time, the difficulty of achieving cure increases, and recurrence rates continue to rise. Notably, the proportion of patients requiring repeat surgery due to local recurrence is 9% for localized TGCT and 23% for diffuse TGCT. Recurrent TGCT following multiple surgeries may lead to significant or complete loss of joint function, secondary joint disease, and potentially necessitate joint replacement (14).

Among the included literature, 45 studies addressed treatment strategies, with non-surgical or combined therapies accounting for 42.2% (19 articles) of treatment-related research. This highlights the growing interest in non-surgical approaches to TGCT recurrence, although nearly all studies acknowledge the essential and irreplaceable role of surgery. Surgical treatment-focused studies comprised 13.5% (12 articles), emphasizing that surgery remains primarily indicated for patients unable to tolerate or unsuitable for non-surgical interventions. Therefore, while non-surgical strategies continue to be explored, there remains a need for clinical investigators to optimize surgical approaches. Moreover, the timing of conservative treatment and the application of non-surgical therapies are emerging as potential future research directions.

To elucidate the underlying mechanisms of TGCT recurrence, 5.2% (7 articles) of studies focused on molecular pathways, aiming to overcome limitations of current treatment modalities. Although the proportion of mechanistic research is relatively low, it provides the most direct evidence for guiding drug development and highlights the current paucity of effective therapies. Additionally, 14.1% (19 articles) examined non-surgical interventions, including radiotherapy, chemotherapy, and targeted therapy, while 12.6% (17 articles) investigated clinical characteristics and diagnostic features of TGCT, informing treatment decisions, precise diagnosis, and disease control. Prognostic factor analyses accounted for 13.4% (18 articles), revealing critical determinants such as tumor subtype, size, location, completeness of surgical excision, and tumor biology. Early diagnosis and personalized treatment planning, facilitated by multidisciplinary teams, are crucial for optimizing surgical and systemic management. Increased engagement from researchers in this field is warranted, as it is likely to become a primary focus of TGCT research.

A detailed review of the ten most-cited articles shows that they primarily address various multimodal treatment strategies. While acknowledging the effectiveness of surgery, these studies advocate for adjuvant therapies to prevent and control recurrence. Adjuvant treatments were consistently reported to reduce recurrence and improve overall patient survival, particularly in cases with unresectable tumors or tumors located in regions where surgery may result in amputation or severe functional impairment. The most-cited study has guided FDA-approved treatment protocols and holds a prominent position in TGCT therapeutics. Of the top ten cited papers, seven were clinical studies and three were reviews, with non-surgical or multimodal therapy studies comprising 70% (7 articles), further underscoring the research emphasis on these approaches.

Our bibliometric analysis also revealed that Cureus Journal of Medical Science contributed the largest number of publications (6 of 134), followed by Skeletal Radiology (4), Case Reports in Orthopedics (3), and Frontiers in Oncology (3). These journals encompass both specialized orthopedic publishers and multidisciplinary platforms, and most TGCT recurrence studies were published in clinical journals, with relatively few reports in basic science outlets. This further highlights the predominance of clinical case analyses over mechanistic investigations. Notably, one of the publishers specializes in oncologic imaging, indicating ongoing efforts to explore the role of imaging in TGCT diagnosis, treatment planning, and prognosis. Among the included articles, 47 (35.1%) were case reports. Authors represented 238 distinct institutions, and thematic classification suggests that, due to the rarity and low regional incidence of TGCT, most institutions report isolated cases rather than conduct systematic investigations.

The United States and China were identified as leading countries in TGCT recurrence research, with the US ranking first. Approximately 60% of the top ten institutions were located in the US, followed by China and Italy. Close collaborations were observed among the US, China, Italy, Japan, and Finland, with the UK also actively collaborating with the US, China, and Italy. Despite this, the breadth and depth of international collaboration remain limited, particularly between US and Chinese institutions, which may impede long-term development in this field. Strengthened international and inter-institutional collaboration is recommended to accelerate the development of effective TGCT recurrence management strategies.

This study represents the first systematic bibliometric analysis of TGCT recurrence, providing comprehensive guidance for researchers. The use of VOSviewer ensured objective and reproducible analysis, enabling a more complete understanding of research hotspots and emerging trends compared to traditional narrative reviews. Limitations include reliance on the Web of Science Core Collection, potentially missing publications indexed in other databases, and the exclusion of letters, conference papers, and patents, which may slightly affect data completeness. Nonetheless, these limitations do not alter the primary trends identified. Overall, this study establishes a foundation for understanding the themes, hotspots, and developmental trajectory in TGCT recurrence research.

## Conclusion

This study systematically reviewed 134 publications on the recurrence of tenosynovial giant cell tumor (TGCT) retrieved from the Web of Science Core Collection. In addition to establishing a foundational research framework for this field, the study precisely identified the key value and future directions of TGCT recurrence research. Due to the tumor’s low global incidence and predominantly sporadic case distribution, individual medical centers often report limited case numbers. Researchers have suggested that this geographic and epidemiologic characteristic may be one of the main reasons for the relative scarcity of studies on TGCT recurrence.By establishing multi-institutional collaborative research networks and expanding tumor sample sizes, research efficiency can be significantly enhanced, potentially facilitating the development of more effective treatment strategies. Furthermore, this study provides potential collaborators for researchers in the field. Importantly, a literature retrieval system for TGCT recurrence was developed, enabling rapid access to cutting-edge research findings and substantially reducing the time required for knowledge acquisition. The analysis also revealed existing gaps in the literature, highlighted emerging trends, and outlined directions for future research, offering valuable guidance for both subsequent investigations and clinical practice.

## Data Availability

The original contributions presented in the study are included in the article/supplementary material. Further inquiries can be directed to the corresponding authors.
